# Physiological, morphological and ecological traits drive desiccation resistance in north temperate dung beetles

**DOI:** 10.1186/s40850-021-00089-3

**Published:** 2021-09-09

**Authors:** Beatrice Nervo, Angela Roggero, Dan Chamberlain, Enrico Caprio, Antonio Rolando, Claudia Palestrini

**Affiliations:** Department of Life Sciences and Systems Biology, University of Torino, Via Accademia Albertina 13, 10123 Torino, Italy

**Keywords:** Climate change, Drought, Effect traits, Nesting behaviour, Phylogenetic signal, Response traits, Scaled body mass

## Abstract

**Background:**

Increasing temperatures and changes in precipitation patterns threaten the existence of many organisms. It is therefore informative to identify the functional traits that underlie differences in desiccation resistance to understand the response of different species to changes in water availability resulting from climate change. We used adult dung beetles as model species due to their importance to ecosystem services. We investigated: (i) the effect of physiological (water loss rate, water loss tolerance, body water content), morphological (body mass) and ecological (nesting behaviour) traits on desiccation resistance; (ii) the role of phylogenetic relatedness in the above associations; and, (iii) whether relatively large or small individuals within a species have similar desiccation resistance and whether these responses are consistent across species.

**Results:**

Desiccation resistance decreased with increasing water loss rate and increased with increasing water loss tolerance (i.e. proportion of initial water content lost at the time of death). A lack of consistent correlation between these traits due to phylogenetic relatedness suggests that the relationship is not determined by a shared evolutionary history. The advantage of a large body size in favouring desiccation resistance depended on the nesting behaviour of the dung beetles. In rollers (one species), large body sizes increased desiccation resistance, while in tunnelers and dwellers, desiccation resistance seemed not to be dependent on body mass. The phylogenetic correlation between desiccation resistance and nesting strategies was significant. Within each species, large individuals showed greater resistance to desiccation, and these responses were consistent across species.

**Conclusions:**

Resistance to desiccation was explained mainly by the dung beetles’ ability to reduce water loss rate (avoidance) and to tolerate water loss (tolerance). A reduction in water availability may impose a selection pressure on body size that varies based on nesting strategies, even though these responses may be phylogenetically constrained. Changes in water availability are more likely to affect dweller species, and hence the ecosystem services they provide.

**Supplementary Information:**

The online version contains supplementary material available at 10.1186/s40850-021-00089-3.

## Background

Declines in insect abundance, biomass, and range are being reported worldwide, across insect orders, and from a spectrum of ecological guilds (e.g., [1, 2]). Increasing temperatures and changes in precipitation patterns represent two of the main threats to the persistence of many insect species [3], with low to mid latitude populations being most at risk [4]. Precipitation patterns are expected to become more variable under climate change, therefore species may have to tolerate longer periods between precipitation events, and the events themselves may be more extreme [5, 6].

Species and populations can respond to climate change by shifting their distribution and tracking optimum environments. However, in the absence of suitable habitat, or the capacity to relocate, species must adapt or go extinct [4]. Furthermore, marked species turnover could strongly affect ecosystem functioning [3, 7]. In grazed ecosystems, for example, dung beetles (Scarabaeoidea: Scarabaeidae, Aphodiidae, Geotrupidae) provide several ecosystem functions and services through the manipulation of livestock faeces for feeding and nesting processes [8–13]. In the Mediterranean area, characterized by high levels of dung beetle diversity and endemism [14–16], increasing temperature due to climate change is expected to induce a northward shift of thermophilous species (e.g. Scarabaeidae), more adapted to live in arid and warm conditions, that may replace the activity of mesophilous dung beetles that live in less extreme conditions (Aphodiidae, Geotrupidae). This rapid replacement of species is especially worrying at the southernmost extremities of Europe (e.g. the Italian peninsula) where the influx of a more thermophilous fauna would be probably more difficult because of the distance from the African continent [17]. Moreover, the effects of climate change on species’ distributions may also be influenced by the immature phases of a dung beetle’s life cycle (i.e. larvae, pupae) that might be even more sensitive to desiccation. Under these conditions, the distribution of resources—e.g. the extent and distribution of dung and suitable habitats—will be a key factor limiting species’ responses to climate change. The dramatic abandonment of extensive historical grazing land, habitat degradation and use of medical veterinary products represent serious additional threats to dung beetle distribution changes [18].

Research effort with regards to climate change has mostly been focused on the consequences of increasing temperatures [19]. However, changes in water availability will also play a significant role [20], especially because it is known to be associated with insect distributions [7, 21] and is implicated, across taxa, in generating stronger selection gradients than temperature [22]. Although the complexity of natural systems presents fundamental limits to predictive modelling, understanding the interactions between environmental stressors and response traits, identifying which traits underpin a failure to adapt, and determining whether response traits change across species, can provide a useful first approximation as to how species will respond to climate change [23]. The effects of changes in water availability are mediated through physiological, morphological, and behavioural responses of organisms [7, 24], thus the relationship among traits that drive these responses needs to be investigated. Physiological adaptations to desiccation resistance (i.e. the ability to resist losing water, measured as survival time) and the underlying traits (e.g. *water loss rates* – proportion of body water lost per hour, *water loss tolerance* – proportion of initial water content lost at the time of death, and *water content* – maximum water content stored) are suggested to be key traits under selection [25, 26], and their study will allow the understanding of how species will survive dry environments [27] through mechanistic models. In this context, dung beetles represent an excellent model group for the kind of physiological investigations required for such models [28–30], and they also represent an under-utilized opportunity to understand the relationship between morphological, ecological and physiological variation at intraspecific, interspecific and assemblage levels [30, 31].

Recent studies using trait-based approaches have shown the significance of traits such as body mass and nesting behaviour as response traits (i.e. traits related to individual fitness), in addition to effect traits (i.e. traits that impact on ecosystem functions) [32, 33]. Dung beetle species show different feeding and nesting strategies, e.g. tunnelers dig tunnels below the dung mass in which they bury brood balls, dwellers brood their young inside the dung-mass itself, or at the soil–dung interface, and rollers transport dung balls some distance away from the dung pat before burial below the soil surface. The action of these different groups has been shown to have a complementary effect in space and time on dung removal rates and other functions such as bioturbation, nutrient cycling, plant growth enhancement and greenhouse gas emissions [9, 12, 13, 34–36], with larger species having a disproportionate effect on many ecosystem functions [9, 37]. At the same time, nesting behaviour and body mass also represent important response traits to environmental variables such as water availability or temperature (e.g., [38–40]). The interplay between response traits and physiological traits requires more attention in dung beetle ecology, especially the role of variations in body water content and water loss rates in modulating desiccation resistance, and how these variables, in turn, are related to size or behavioural traits like nesting strategies [30]. The literature on this topic is mainly focused on African dung beetle communities [30], while, to our knowledge, little effort has been made in north temperate areas.

In this study, we used adult dung beetles to investigate: (i) physiological (i.e. water loss rate, water loss tolerance, water content), morphological (i.e. body mass), and ecological (i.e. nesting behaviour) traits that drive desiccation resistance; (ii) the role of phylogenetic relatedness in the above associations; and (iii) whether the effect of relative size within species on desiccation resistance is consistent across species or whether it is modulated by species identity. We expected that desiccation resistance would be mainly affected by low water loss rates and high water loss tolerance, but also by larger body sizes that reduce area-to-volume ratio [38, 41], thus improving water conservation [39]. We also expected that different nesting behaviours would result in species that may experience different environments and respond differently to water availability, with tunnelers and rollers being more tolerant to arid conditions.

## Results

### General effects model

Desiccation resistance was modulated both by physiological (i.e. water loss rate, water loss tolerance), morphological (i.e. body mass), and ecological (i.e. nesting behaviour) traits.

**Fig. 1:**
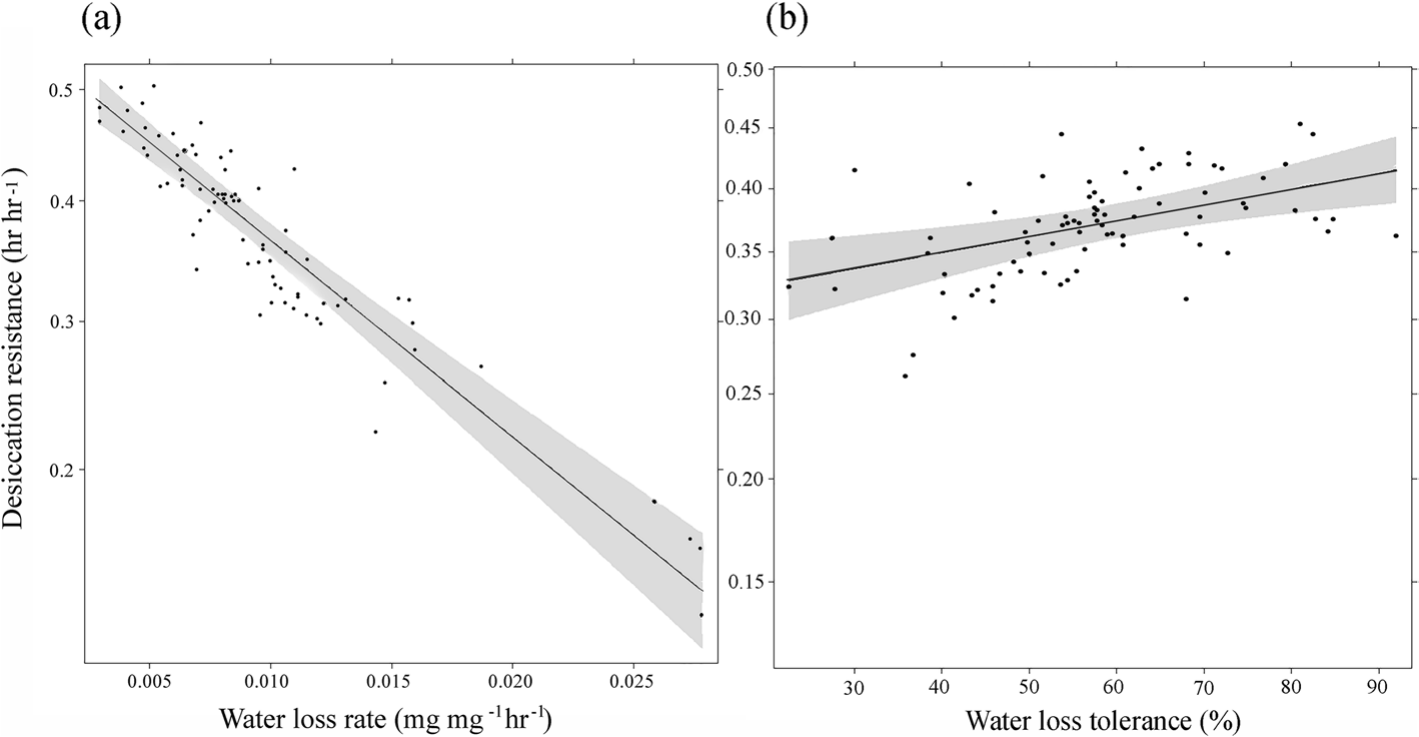
Desiccation resistance of dung beetles is influenced by water loss rate, expressed as proportion of initial water content lost per hour (**a**), and water loss tolerance, expressed as the proportion of the initial water content that was lost at the time of death (**b**). 95% confidence bands are displayed around each fitted line

**Fig. 2:**
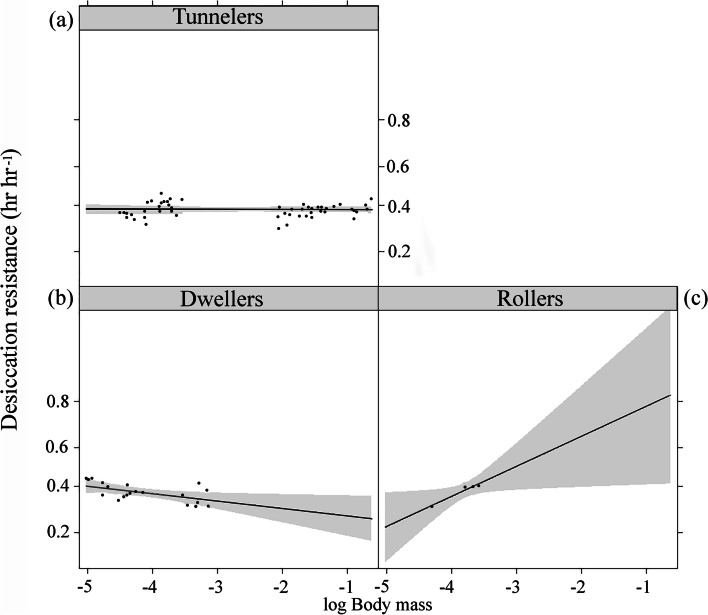
Desiccation resistance is influenced by the interaction effect between body mass and nesting strategies: tunnelers (**a**), dwellers (**b**), and rollers (**c**). 95% confidence bands are displayed around each fitted line

Desiccation resistance was explained by water loss rate, water loss tolerance, body mass (log), and nesting strategies. Desiccation resistance decreased with increasing water loss rate and increased with increasing water loss tolerance (Fig. 1). We found a significant interaction between body mass and nesting behaviour: desiccation resistance in rollers increased linearly with body mass, while it was not influenced by body mass in tunnelers and gradually decreased with body mass in dwellers (Table 1, Fig. 2).

**Table 1:** Associations among physiological, reproductive and morphological traits

**GLMER (sample size = 80, species = 8)**			
DR ~ NS * log BM + WLR + WLT + random factor (species), distribution = Binomial			
AIC = 506.3			
Variance explained = 0.89			
	Estimate ± SE	z value	*p*
Intercept	-0.796 ± 0.287	-2.777	**
Rollers	3.081 ± 1.181	2.614	**
Tunnelers	0.680 ± 0.285	2.380	*
Log BM	-0.155 ± 0.068	-2.292	*
WLR	-71.831 ± 5.372	-13.355	***
WLT	0.005 ± 0.001	3.558	***
Rollers * log Body mass	0.785 ± 0.308	2.554	*
Tunnelers * log Body mass	0.156 ± 0.069	2.245	*

*DR* Desiccation Resistance is related to *WLR* Water Loss Rate, *WLT* Water Loss Tolerance, *BM* Body Mass, and *NS* Nesting Strategy. Fractional water content is not shown in the model because it was not significant. *SE* Standard Error. * *p* < 0.05; ** *p* < 0.01; ** *p* < 0.001

The phylogenetic signal, defined by the variance of the standardized contrast values (VarContr, Table S1 in Additional file 2) was significant for desiccation resistance (DR = 0.011), water loss rate (WLR = 0.000), and nesting strategies (NS = 0.357), while it was not significant for the other traits (Table S1 in Additional file 2). Phylogenetic independent contrasts (PicR) between desiccation resistance and the other traits showed a significant correlation between desiccation resistance and nesting strategies (*p* = 0.001, Table S2 in Additional file 2), suggesting that the hypothesized relationship between these two traits may also be determined by the evolutionary history of species.

### Relative effects model

**Fig. 3:**
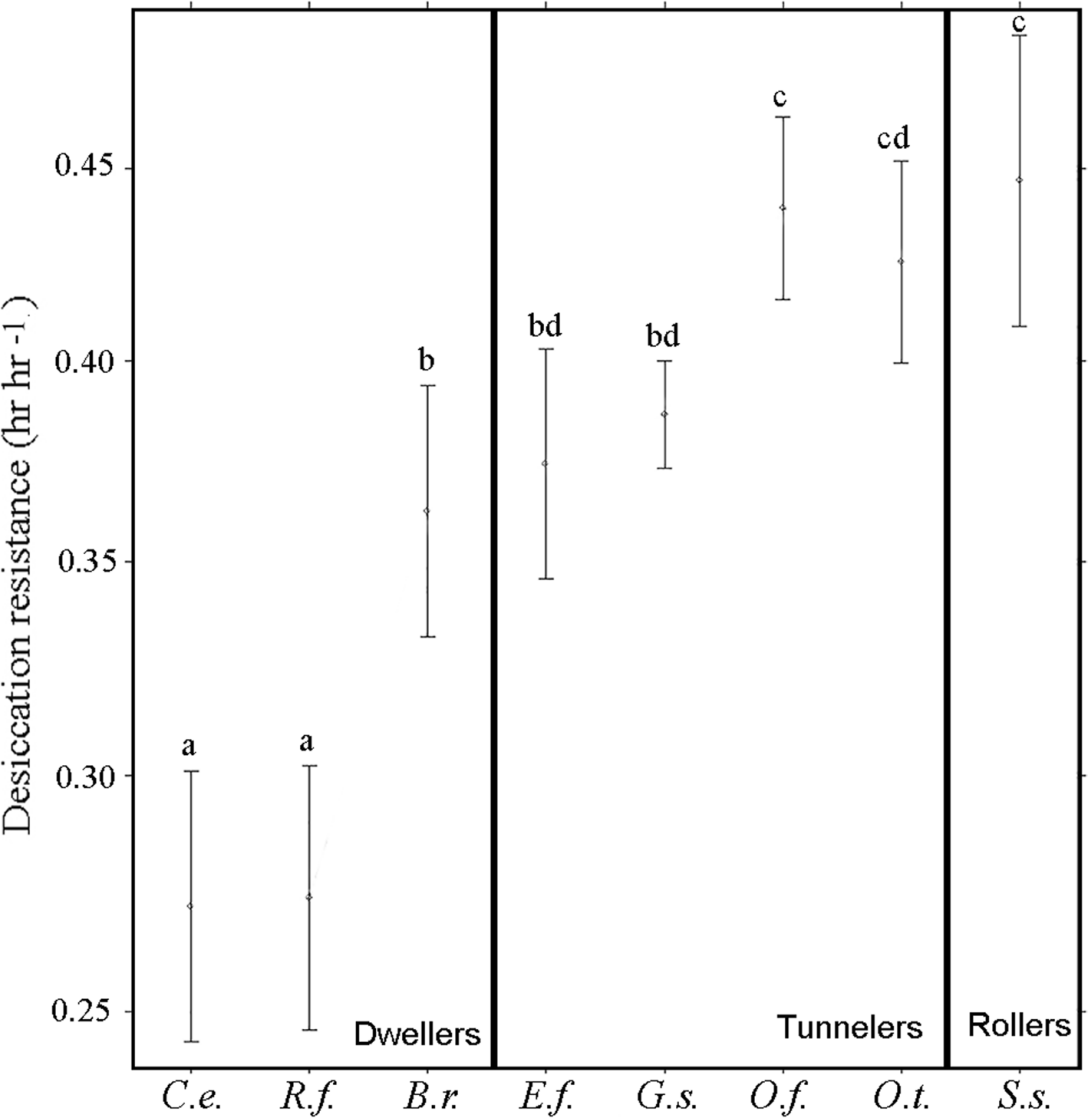
Differences in desiccation resistance among the eight species. Letters over the error bars indicate the differences among species. Species have been grouped based on their nesting strategies into dwellers, tunnelers, and rollers. Abbreviations for species names means: *C.e.* = *C. erraticus*, *R. foetens* = *R. f*, *B.r.* = *B. ruf*a, *E.f.* = *E. fulvus*, *G.s.* = *G. stercorarius*, *O.f.* = *O. fracticornis*, *O.t.* = *O. taurus*, *S.s.* = *S. schaefferi*

Desiccation resistance depended on species, scaled body mass, scaled water loss rate, and scaled water loss tolerance (scaled variables were expressed using the respective average value for each species, see Methods for further details). Within each species, large individuals were more resistant to desiccation than smaller ones due to lower rates of water loss and higher water loss tolerance. Species significantly differed in desiccation resistance, with *O. fracticornis*, *O. taurus*, and *S. schaefferi* showing higher levels of resistance compared to the other species (Table 2, Fig. 3).

**Table 2:** Interspecific and intraspecific differences in desiccation resistance

**GLM (sample size = 80, species = 8)**			
DR ~ scaled WLR + scaled WLT + scaled BM + Species, distribution = Binomial			
AIC = 497.97			
Variance explained = 0.87			
	Estimate ± SE	z value	*p*
Intercept	-0.971 ± 0.073	-13.267	
Scaled WLR	-0.734 ± 0.063	-11.682	***
Scaled WLT	0.411 ± 0.101	4.070	***
Scaled BM	0.163 ± 0.066	2.454	*
*R. foetens*	0.009 ± 0.102	0.092	NS
*B. rufa*	0.421 ± 0.099	4.234	***
*E. fulvus*	0.472 ± 0.096	4.891	***
*G. stercorarius*	0.524 ± 0.079	6.623	***
*O. fracticornis*	0.744 ± 0.089	8.337	***
*O. taurus*	0.687 ± 0.915	7.507	***
*S. schaefferi*	0.773 ± 0.109	7.075	***

*DR* Desiccation Resistance is related to *WLR* Water Loss Rate, *WLT* Water Loss Tolerance, and *BM* Body Mass. Fractional water content is not shown because it was not significant. *C. erraticus* is the reference level. *SE* Standard Error, *NS* Not Significant. * *p* < 0.05; ** *p* < 0.01; ** *p* < 0.001

Since rollers were represented by only one species, we compared general and relative models that included *S. schaefferi* with those that did not include this species. The general and relative models that did not include *S. schaefferi* were qualitatively the same as the models including this species, thereby supporting the consistency of the results and confirming that the inclusion/exclusion of this species did not affect our general conclusions (see models without *S. schaefferi* in Tables S1 and S2 in Additional file 1).

## Discussion

A trait-based approach integrated with mechanistic physiological research and assemblage-level ecological studies can contribute not only to a better understanding of an ecologically important group such as dung beetles, but also to predicting the functional consequences of community changes on ecosystems. Our results provide support to our hypothesis that suggests water loss rates and water loss tolerance as important traits driving desiccation resistance in dung beetles. They also showed evidence that species with different nesting behaviours have different abilities to resist desiccation. The effect of large body mass in enhancing water conservation under stress conditions was especially evident at an intraspecific level.

### Effects of physiological, morphological and ecological traits on desiccation resistance

Species distributions are often constrained by climatic tolerances, but the ability of species to adapt to changing environments may also be constrained by their evolutionary history [42]. Despite the limited number of species analyzed, the significant phylogenetic signal found in our study for desiccation resistance and water loss rate suggests that physiological mechanisms to reduce water loss and increase resistance may be highly dependent on species’ life histories.

Trait correlations due to phylogenetic relatedness may play an important role in shaping ecological adaptation [43]. However, a lack of consistent trait correlation between desiccation resistance, the three physiological traits (i.e. water loss rate, water loss tolerance, and fractional water content) and body mass suggests that the relationships between these traits are not strictly dependent on phylogenetic relatedness between the species [43].

Our study showed that the resistance of the investigated dung beetles to desiccation is explained mainly by their ability in reducing water loss rate (avoidance) and tolerating water loss (tolerance), as also shown in previous research [44, 45]. Here, water loss rate was shown to better predict desiccation resistance than water loss tolerance and water content. These results confirmed previous research on other model organisms (e.g. isopods, [46]) that showed how water loss rate is the main factor explaining resistance to desiccation. Moreover, we found that, after a certain percentage of water loss (about 50%), a dung beetle’s capability to resist desiccation decreased significantly. This percentage of water loss, that corresponds to reduced survival, seems to reflect a common pattern in arthropods; for example, fatal water loss is between 40 and 55% for isopods, ants, and weevils [46–48], even though it can show higher values for other detritivorous taxa (e.g. earthworms 70–75%, [49]). In our study, some tunneler species belonging to the Scarabaeinae (e.g. *O. fracticornis*, *O. taurus*, *E. fulvus*), showed a high percentage of water loss tolerance (64–70%). The high values of water loss tolerance in these species indicates greater resistance to possible frequent dry periods under climate change.

Our study showed that body mass and nesting strategies are important morphological and ecological traits that influence desiccation resistance in adult dung beetles. Body mass has also been shown to be a key trait in influencing water balance strategies among species in other taxa, e.g. weevils, keratin beetles, isopods, flies [28, 47, 50, 51, 52]. Previous studies have shown that larger body size reduces the surface area-to-volume ratio [38, 41], thus improving water conservation. This may represent an advantage in terms of a reduced water loss rate [39], since body area does not increase linearly with body mass or volume. In line with previous studies, at an intraspecific level (i.e. relative effect models), large individuals seemed to be more resistant than small individuals and these responses were consistent across species. The intraspecific variability between individuals is at the base of natural selection and is important for the adaptation of organisms to environmental changes. However, when the effect of body mass on desiccation resistance was evaluated across species (i.e. general effect models), we found a significant interaction effect between body mass and functional groups based on nesting behaviour, suggesting that changing conditions in water availability of soil, dung or air may impose a selection pressure on body size that will vary in groups with different nesting strategies. In rollers, represented by only one species, large body sizes increased desiccation resistance, while in tunnelers and dwellers, desiccation resistance seemed not to be dependent on body mass. In tunnelers, the effects of large size in increasing desiccation resistance were not particularly evident, since large species such as *G. stercorarius* were characterized by lower desiccation resistance compared to smaller species such as *O. fracticornis*. In dwellers, we found a very slight tendency for desiccation resistance to increase with body mass which needs further investigation. These results may be explained by the fact that traits characterizing an individual can interact in complex, and sometimes opposite, ways by causing potential trait-specific constraints [24, 53]. For example, the advantageous effect of large body sizes with respect to resistance to desiccation may be counterbalanced by other traits that favour water loss. Differences in water loss rate may be linked to anatomical and morphological characteristics such as spiracle size, position and number which are highly variable within the Scarabaeoidea, but that may be more similar in closely related species [54]. The ability of insects to conserve water and resist desiccation is also influenced by the movement of gas and water through the system of spiracular openings and tracheae [55] that may affect respiratory water loss.

Nesting strategy seemed to influence the physiological variations among the investigated species. The tunneler and roller species used in our study were generally more resistant to desiccation than dwellers because of their lower water loss rates and water loss tolerance. It is worth noting that the more resistant tunnelers belonged to the Scarabaeidae family, while dwellers belonged to the Aphodiidae family. Despite phylogenetic relatedness that may explain part of this relationship, species characterized by different nesting behaviours are more likely to experience different environments that lead to different responses to dry conditions. Dwellers spend most of their time inside the dung pat or in the dung-soil interface, where the humidity level is high (about 80% RH), whereas tunnelers feed on dung, but they spend much time in the soil where they dig tunnels to lay their eggs. Larvae may also play an important role in determining different responses between nesting strategies: larvae in dung-ovipositing species may be more exposed to desiccation than soil-ovipositing species whose eggs are laid underground and inside dung-compacted reproductive balls. Future in-depth analyses considering a larger number of species and different life-cycle stages are crucial for making predictions about species responses to climate changes.

Our study showed that species belonging to Scarabaeidae such as *O. fracticornis*, *O. taurus*, and *S. schaefferi* are the most resistant to desiccation. In accord with [17], these species are more likely to respond to climate change by shifting their distributions northward and reaching new territories. However, [17] showed that *O. taurus* is expected to increase its current distribution under global warming, while *O. fracticornis* and *S. schaefferi* are likely to lose part of their current distribution.

### Caveats on interpretation

There are a number of caveats that need to be made when interpreting the results. First, the water-related traits were measured gravimetrically in order to follow a standardized protocol [56] to enable comparative analysis. However, water in insects can be lost in different ways (i.e. transpiration through the cuticle, evaporation along open spiracles through the tracheal system, and excretion – [39, 44]), and our approach does not take into consideration the different mechanisms underlying specific adaptation to reduce water loss pathways in different species. Second, we controlled water loss through excretion by starvation, as also suggested by the acclimation procedure in [56]. However, we should highlight that starvation may decrease metabolic rates, resulting in changes to cuticle and respiratory water loss [57]. Different metabolic rates and activities of insects during the experiment vary at the species level which is an extra-factor that may cause interspecific differences. This aspect can be taken into account in future studies, and appropriate alternatives to the acclimation procedure can be carried out by implementing the protocol in [56]. Third, we considered 70% as a relevant desiccation stressor for dung beetles since 85% humidity has been suggested to be an appropriate RH for insects inhabiting soil [56, 58]. However, dung beetles are flying arthropods that for short periods can be exposed to lower humidity. For this reason, future studies should test different levels of humidity encompassing values from 0 to 70% to assess consistency of responses under different experimental conditions.

### Ecological implications

Changes in community structure, and turnover of dung beetle species and functional traits may have consequences for the provisioning of ecosystem functions and services. Body size and nesting strategy have been shown to be crucial response traits in dung beetles [32, 33] that make species more prone to extinction, but at the same time they represent effect traits that influence the provisioning of ecosystem services such as soil nutrient cycling, dung removal and herbage growth (e.g. [9, 12, 13, 33]). In our study, roller and tunneler species seemed to be the most resistant to desiccation, suggesting that changes in patterns of water availability, under a climate change context, probably will influence mostly dweller species with dung and soil-ovipositing nesting strategies. By spending most of their time inside the dung pat, dwellers may be more influenced by physical factors affecting dung, and this can explain the preference for closed habitats in these species [59].

Dwellers represent the main functional group in some areas such as the Alps, and even if their functional effect on dung removal, herbage growth and nutrient cycling seems less marked over a short time period compared to tunnelers, the tunnelers and dwellers seem similarly efficient for most functions, with differences based on the spatial and temporal scales over which the functions operate [12]. The replacement of species sensitive to water limitation (e.g. dwellers) by more resistant species (e.g. tunnelers or rollers) under a warming climate, may influence the provisioning of ecosystem services, especially if larger sized species, which have a relevant effect on ecosystem functioning, are more prone to local disappearance. Furthermore, warming temperature and lower humidity have been shown to decrease brood production and dung burial by tunnelers and rollers (e.g. *Onthophagus taurus* and *Sisyphus rubrus*) suggesting the impactful effect of climate change on the provisioning of ecosystem services [60, 61], but see [62]. This trait-based analysis raises questions for the direction of future research, for example, regarding possible ecological and evolutionary implications of the interactions between changes in water availability and warming temperatures, potential behavioural changes of tunnelers, rollers and dwellers in response to reductions in moisture conditions, and how these changes will affect trophic interactions.

## Conclusions

In conclusion, resistance to desiccation was explained mainly by the dung beetles’ ability to reduce water loss rate (avoidance) and to tolerate water loss (tolerance). Larger individuals within each species were more resistant than smaller individuals and these responses were consistent across species. However, when the effect of body mass on desiccation resistance was evaluated across species, we found a significant interaction between body mass and functional groups based on nesting behaviour, suggesting that changing conditions in water availability may impose a selection pressure on body size that varies in groups with different nesting strategies, even though these responses may be phylogenetically constrained. Changes in water availability are more likely to affect dweller species, and hence the ecosystem services they provide.

## Methods

### Species collection and trait measurements

Adult individuals of 8 different dung beetle species were collected in September 2019 in four different areas in the north-west of Italy (Piedmont) (Figure S3 in Additional file 1). Five species were collected from the pastures of the Istituto per le Piante da Legno e l’Ambiente (IPLA) in Torino (45°05′18.5′′N, 7°44′28.5′′E, 300 m a.s.l), one species from the pastures around Foresto (45°08′32″N 7°07′14″E, Susa Valley, 470 m a.s.l.), four species from the pastures around Demonte (44°18′59′′N, 7°17′59′′E, Stura Valley, 780 m a.s.l.), and two species from the high altitude pastures around the sanctuary of S. Anna di Vinadio (44°13′55.2″N, 7°06′18″E, Stura Valley, 2035 m a.s.l.). Four species were present in more than one area (*G. stercorarius* and *O. fracticornis* in Torino and S. Anna di Vinadio*; O. taurus* and *E. fulvus* in Torino and Demonte). Species, collected mainly in cattle dung, represented three different functional groups defined according to nesting behaviour: tunnelers, dwellers, and rollers (Table 3, to note that only one species belonged to rollers). These species are commonly found in cattle dung, but they are able to utilize a wide range of dung coming from different herbivorous mammals such as horse and sheep dung [63]. Sex ratio was approximately 50:50 for each species.

**Table 3:** Species collected in the four sampling areas

**Family**	**Species**	**Nesting strategy**	**Sampling areas**			
			**Torino**	**Foresto**	**Demonte**	**S. Anna di Vinadio**
Geotrupidae	*Geotrupes stercorarius (Linnaeus, 1758)*	Tunneler	8			30
Scarabaeidae	*Onthophagus fracticornis (Preyssler, 1790)*	Tunneler	9			30
	*Onthophagus taurus (Schreber, 1759)*	Tunneler	9		7	
	*Euoniticellus fulvus (Goeze, 1777)*	Tunneler	7		8	
	*Sisyphus schaefferi (Linnaeus, 1758)*	Roller		8		
Aphodiidae	*Rhodaphodius foetens (Fabricius, 1787)*	Dweller	7			
	*Colobopterus erraticus (Linnaeus, 1758)*	Dweller			7	
	*Bodilopsis rufa (Moll, 1782)*	Dweller			7	

Dwellers include both dung- and soil-ovipositing species. Dweller species with a soil-ovipositing behaviour are *C. erraticus* [64, 65]. The number of individuals of each species used in the experiment is specified. The overall sample size was 137 individuals

We followed the trait-based protocol suggested by [56] for standardized measurement of traits in terrestrial invertebrates. The explicit guidelines provided by the authors have the potential to serve as a basis for comparative studies using functional traits [66]. Before exposing beetles to dry conditions and measuring desiccation resistance, individuals were acclimatized by replenishing any possible water deficit in order to start the experimental measurements with approximately the maximum possible body water content. This pre-treatment procedure was done without food to induce animals to empty their gut and reduce faecal production, in order to avoid any change in body mass not related to water loss. Beetles were kept isolated in small cylinders (diameter 2 cm, height 3 cm) placed in a closed glass box (40 × 20 × 20 cm), on top of a 3 cm layer of moist floral foam for 3 days, ensuring constant conditions of 100% relative humidity (RH). The level of humidity inside the box was measured with humidity data loggers (Plug & Track™). The laboratory was kept at a temperature of 20 °C for the whole acclimation period (average temperature: 20.4 ± 0.3 °C) under a12:12 h photocycle. The cylinders were open at both sides which were covered with a nylon mesh cloth (width 0.5 mm) to prevent beetles from escaping, but allowing an adequate airflow in the cylinder. Based on the abundance of each species, one, two or three individuals were kept in the glass container (100% RH) after the starvation period as a control; none of these controls died before the end of the desiccation resistance measurements.

Dung beetles were exposed to moderate dry conditions, approximately 70% RH (average value: 69.4 ± 1.3%), to record survival time, water loss rate, and percentage of fatal water loss. This RH value was chosen to represent a moderate stress condition considering that, on average, cattle dung has a relative humidity of 80%, and that 91–93% is the threshold above which arthropods (e.g. terrestrial isopods, beetles) are able to absorb water vapour [67, 68]. The humidity level of 70% RH was reached using a glycerol–water solution in volume concentration of 48% (as per [46]. The level of humidity inside the cylinders was measured with humidity data loggers. Plastic glasses (200 ml) were filled with 80 ml of glycerol solution. In each glass, a platform made of steel wire was placed about 1 cm above the solution’s surface; the cylinder containing the animal was then placed on this platform and the glass closed with a plastic cover. The platform allowed air exchange between the solution and the cylinder inside the glass. The temperature range for the experimental period was 21–22 °C.

We measured 7–39 individuals per species depending on the abundance of the animals collected in the field (Table 3). Before exposing the beetles to dry conditions, individuals in each cylinder were weighed to record their initial fresh mass using an analytical balance (Precisa 125 A, 0.1 mg). We recorded changes in individual body mass (mg) every 3 h during the day (9:00 am, 12:00 am, 3:00 pm, 6:00 pm). Before weighing the dung beetles, we checked if they were alive by disturbing them gently with a soft brush or by flipping them with tweezers. By weighing the cylinders containing the beetles at regular times, we were able to register weight changes, and, at the same time, minimize disturbance, as also shown by [46]. The test was conducted for 96 h, after which we ended the experiment even if some individuals were still alive, to avoid other potential stress factors, such as starvation, influencing the measurements [56]. Animals that were still alive at the end of the experiment were killed by exposing them to ethyl acetate, an efficient method used in entomology [69]. Dead animals were weighed and frozen for further morphological analyses.

We measured desiccation resistance and the three underlying physiological traits (i.e. water loss rate, fatal water loss, and water content) only on individuals that died during the experiment (Table 4). *Desiccation resistance* was estimated as survival time, which refers to the time that dry conditions can be tolerated before an organism dies. Survival time of each individual was calculated as the number of hours an organism survives in proportion to the total number of hours of the experiment, expressed as a value between 0 (0 h $/$ 96 h) and 1 (96 h/96 h). If individuals died overnight, the median of the values (in hours) of the last measurement in the afternoon (e.g. 6:00 pm) and the first in the morning (e.g. 9:00 am) was used to calculate desiccation resistance, according to [56]. *Water loss rate* describes the rate of water loss from an individual over a given period of time (proportion of initial water content lost per hour); it was estimated as the slope of the linear regression between water mass and time and expressed as the proportion of initial body water content that was lost per unit of time (mg mg^−1^ h^−1^) [46]. *Water loss tolerance* was expressed as the proportion of the initial water content that was lost at the time of death, i.e. [(initial wet body mass $-$ final wet body mass)$/$ initial water content] $\times$ 100. If the individual died overnight, we used the average value of body mass of the last measurement in the afternoon and the first in the morning. Water content, which is the maximum water content that an organism is able to store, was expressed as a *fractional water content* (initial water content / dry body mass). Potential effects of collinearity among physiological predictor variables were calculated using the pairplot combined with correlation coefficients in R [70]. Water loss rate, water loss tolerance, fractional water content, and body mass were not found to be correlated with each other, allowing us to use them as covariates in the models (Fig. S4 in Additional file 1).

**Table 4:** Average trait values and standard deviations for each species

**Species**	**Dead individuals/Total**	**BM ± SD (mg)**	**DR ± SD (hr hr**^−1^**)**	**WLR ± SD (mg mg hr**^−1^**)**	**WLT ± SD (%)**	**fWC ± SD (mg mg**^−1^**)**
*G. stercorarius*	31/38	247.787 ± 120.305	0.646 ± 0.160	0.008 ± 0.002	50.934 ± 8.631	2.177 ± 3.212
*O. fracticornis*	12/39	18.158 ± 5.334	0.755 ± 0.199	0.007 ± 0.003	70.661 ± 18.170	4.286 ± 1.528
*O. taurus*	8/16	21.175 ± 5.926	0.768 ± 0.193	0.008 ± 0.004	64.465 ± 15.513	2.063 ± 0.694
*E. fulvus*	8/15	18.388 ± 3.966	0.671 ± 0.228	0.012 ± 0.006	69.671 ± 6.255	2.421 ± 1.303
*S. schaefferi*	4/8	22.350 ± 6.267	0.766 ± 0.125	0.005 ± 0.002	47.342 ± 16.704	2.958 ± 1.575
*R. foetens*	7/7	36.643 ± 5.259	0.393 ± 0.145	0.014 ± 0.006	54.812 ± 3.313	2.210 ± 0.495
*C. erraticus*	7/7	8.229 ± 1.497	0.393 ± 0.121	0.018 ± 0.007	62.011 ± 13.101	2.398 ± 0.775
*B. rufa*	7/7	13.014 ± 1.483	0.634 ± 0.126	0.009 ± 0.003	50.173 ± 15.636	2.335 ± 1.620

We specified the number of animals that died during the experiment, *BM* Body Mass, *DR* Desiccation Resistance, *WLR* Water Loss Rate, *WLT* Water Loss Tolerance and *fWC* Fractional Water Content with the respective *SD* Standard Deviations

Once the experiment was over, we measured the dry body mass of each individual. To measure the dry body mass (hereafter body mass), the insects were dried in the oven (Binder FD) at 25 °C for 24 h and weighed until the weight was constant between subsequent measurements (every 12 h).

### Statistical analyses

We used two different statistical approaches: the first was used to investigate morphological, ecological and physiological traits that influence desiccation resistance, while the second was used to test if relatively large and small individuals within species have similar desiccation resistance and whether these responses are consistent across species.

The general effect of physiological (water loss rate, water loss tolerance, and fractional water content), morphological (body mass), and ecological (nesting behaviour) traits on desiccation resistance was tested by using Generalized Linear Mixed effects Models (GLMMs) with a Binomial distribution. Visual inspection of frequency distributions and Shapiro–Wilk tests confirmed the non-normality of errors. Species identity (8 different species) was specified as a random effect to account for non-independence of conspecific individuals, where intercepts were allowed to vary between species. We fitted the models in R package “lme4” [71], with the formula:

$${\text{Desiccation}}\;{\text{resistance}}\; \sim \;{\text{logBody}}\;{\text{mass}}\; * \;{\text{Nesting}}\;{\text{strategy}}\; + \;{\text{Water}}\;{\text{loss}}\;{\text{rate}}\;{ + }\;{\text{Water}}\;{\text{loss}}\;{\text{tolerance}}\;{ + }\;{\text{random}}\;{\text{factor}}\;{\text{(1|Species)}}$$

Henceforth, this is referred to as the general effects model. We checked for interactions and non-linear terms between the covariates. If the interaction effect was not significant, the term was added to the model as an additive factor. We removed non-significant terms from the model (i.e. fractional water content).

In the second approach, we used generalized linear models (GLM) with a Binomial distribution to verify whether desiccation resistance varied in response to individuals with different body size, water loss rate and water loss within each species and if these responses were consistent across species. The variations in body mass, water loss rate and water loss within each species were expressed as scaled values, e.g. (body mass of the individual $-$ average body mass of the species)$/$ average body mass of the species. In this way, we considered how relative intraspecific body mass (i.e. relatively large or small individuals of the same species), water loss rate and tolerance (i.e. individuals of the same species with high or low water loss rate and tolerance) affected desiccation resistance, and whether the within-species pattern was also consistent across species. We modelled desiccation resistance of all individuals as a function of species, scaled body mass, scaled water loss rate and scaled water loss tolerance. Interactions and non-linear terms were initially tested but were not significant, thus all variables were included as additive factors via the formula:

$${\text{Desiccation}}\;{\text{resistance}}\; \sim \;{\text{Species}}\; + \;{\text{Scaled}}\;{\text{body}}\;{\text{mass}}\; + \;{\text{Scaled}}\;{\text{Water}}\;{\text{loss}}\;{\text{rate}}\; + \;{\text{Scaled}}\;{\text{Water}}\;{\text{loss}}\;{\text{tolerance}}$$

Henceforth, this is referred to as the relative effects model. As above, we removed non-significant terms from the model (i.e. fractional water content).

Since only one species belonged to the rollers, we repeated the models for both approaches without *S. schaefferi* to assess whether the inclusion of this species made a difference to the general conclusions.

### Phylogenetic signal and relationships between traits

Fitting a species as a random effect implicitly assumes a constant degree of independence between species (i.e. that of the phylogenetic relatedness is trivial). We assessed the possible effect of phylogeny on our results by constructing a phylogenetic tree based on mitochondrial COI sequence (see Table S3 in Additional file 2 for the list of the accession numbers) and testing the relationship among traits. The COI sequence was chosen as a reliable proxy to define the relationships among coleopteran taxa, according to [72]. Both Maximum Parsimony (MP) and Maximum Likelihood (ML) approaches were used to evaluate the evolutionary history of the species (for the details of the molecular analysis, see Box S4 in Additional file 2). The ML analysis options were set according to the best fit value (Table S5 in Additional file 2), and the evolutionary divergence between sequences was estimated (Table S6 in Additional file 2). The resulting trees were compared and the phylogenetic relationships among the species were examined (Figure S7 in Additional file 2). Then, a reduced matrix was built, in which only a terminal node for each ingroup species was included (Table S3 in Additional file 2) to build a ML tree (Figure S8 in Additional file 2) and to test the phylogenetic signal of the traits. The phylogenetic signal is the tendency of related species to resemble each other more than species drawn at random from the same tree. All the molecular phylogenetic analyses were conducted using MEGA v10 [73].

The phylogenetic signal was calculated using the software PhyloCom v4.2 [74]. In the analysis, a matrix of the mean values of each trait for each species and the reduced ML tree (Fig S8 in Additional file 2) were used to correlate the eight taxa and six traits (i.e., desiccation resistance, water loss rate, water loss tolerance, fractional water content, dry body mass and nesting strategy), based on the phylogenetic relationships of these taxa. A detailed description of the analysis used to calculate the variance of the standardized contrasts (VarContr, Table S1 in Additional file 2) and the phylogenetic independent contrasts (PicR, Table S2 in Additional file 2) can be found in Box S9 in Additional file 2. The PicR method was used to estimate the correlation among traits due to phylogenetic relatedness.

## Supplementary Information

**Additional file 1:** Supporting information on methods. **Table S1.** Associations among physiological, reproductive and morphological traits without including *S. schaefferi *which is the only species belonging to rollers. **Table S2.** Interspecific and intraspecific differences in desiccation resistance without including *S. schaefferi *which is the only species belonging to rollers. **Figure S3.** The four sampling areas where the dung beetle species were collected. **Figure S4.** Pairplot of all response variables.

**Additional file 2:** Phylogenetic analysis results. **Table S1.** The variance of the standardized contrasts (VarContr) value define the amount of phylogenetic signal for each trait. **Table S2.** Independent contrast correlations (traits 2 and higher vs. trait 1). **Table S3.** Accession codes of the COI-5P sequences used in the phylogenetic analysis. The sequences marked by the asterisk were used in the phylogenetic signal analysis. **Box S4.** The phylogenetic analysis. **Table S5.** Maximum Likelihood fits of 24 different nucleotide substitution models. **Table S6.** Estimates of Evolutionary Divergence between Sequences. **Figure S7.** The ML tree with the highest log likelihood (-3191.7887). **Figure S8.** The bootstrap tree from the ML tree with the highest log likelihood (-2731.88). **Box S9.** The Phylogenetic signal.

## Data Availability

The datasets used and/or analysed during the current study are available from the corresponding author on reasonable request.

## Abbreviations

DR: Desiccation resistance
WLR: Water loss rate
WLT: Water loss tolerance
fWC: Fractional water content
NS: Nesting strategies
SE: Standard Error
GLM: Generalized linear models
GLMM: Generalized Linear Mixed effects Models
G.s: *Geotrupes stercorarius*
O.f.: *Onthophagus fracticornis*
O.t.: *Onthophagus taurus*
E.f.: *Euoniticellus fulvus*
R.f.: *Rhodaphodius foetens*
C.e.: *Colobopterus erraticus*
B.f.: *Bodilopsis rufa*
