# Supplementary material for: Physiological, morphological and ecological traits drive desiccation resistance in north temperate dung beetles
Source: BMC Zool. 2021 Sep 9;6:26. doi: 10.1186/s40850-021-00089-3 (PMC10127359; doi:10.1186/s40850-021-00089-3)
Supplement: Supplementary file 1 — Additional file 1: Supporting information on methods. Table S1. Associations among physiological, reproductive and morphological traits without including S. schaefferi which is the only species belonging to rollers. Table S2. Interspecific and intraspecific differences in desiccation resistance without including S. schaefferi which is the only species belonging to rollers. Figure S3. The four sampling areas where the dung beetle species were collected. Figure S4. Pairplot of all response variables. [file 40850_2021_89_MOESM1_ESM.pdf]

Table S1 Associations among physiological, reproductive and morphological traits without including *S. schaefferi* which is the only species belonging to rollers. DR, WLR, WLT, BM, and NS are abbreviations that respectively mean Desiccation Resistance, Water Loss Rate, Water Loss Tolerance, Body Mass, and Nesting Strategy. Fractional water content was not significant. SE = Standard Error.

**GLMER (sample size=76, species=7)**			
DR ~ NS * log BM + WLR + WLT + random factor (species), distribution= Binomial			
AIC= 477.6			
Variance explained= 0.84			
	Estimate±SE	z value	*p*
Intercept	-0.809±0.287	-2.820	**
Tunnelers	0.669±0.286	2.343	*
Log BM	-0.154±0.068	-2.273	*
WLR	-72.721±5.400	-13.466	***
WLT	0.006±0.002	3.749	***
Tunnelers * log BM	0.153±0.069	2.209	*

Table S2 Interspecific and intraspecific differences in desiccation resistance without including *S. schaefferi* which is the only species belonging to rollers. DR, WLR, WLT are abbreviations that respectively mean Desiccation Resistance, Water Loss Rate, Water Loss. Fractional water content was not significant. SE = Standard Error.

**GLM (sample size=76, species=7)**			
DR ~ scaled WLR + scaled WLT + scaled BM + Species, distribution= Binomial			
AIC= 475.09			
Variance explained= 0.88			
	Estimate±SE	z value	*p*
Intercept	-0.972±0.073	-13.278	***
Scaled WLR	-0.759±0.063	-12.022	***
Scaled WLT	0.321±0.106	3.036	**
Scaled BM	0.159±0.067	2.381	***
R. foetens	0.008±0.102	0.078	
B. rufa	0.419±0.099	4.207	***
E. fulvus	0.470±0.096	4.872	***

G. stercorarius	0.525±0.079	6.631	***
O. fracticornis	0.710±0.088	8.039	***
O. taurus	0.685±0.915	7.485	***
S. schaeafferi	0.773±0.109	7.075	***

Figure S3 The four sampling areas where the dung beetle species were collected. The map was created by the authors.

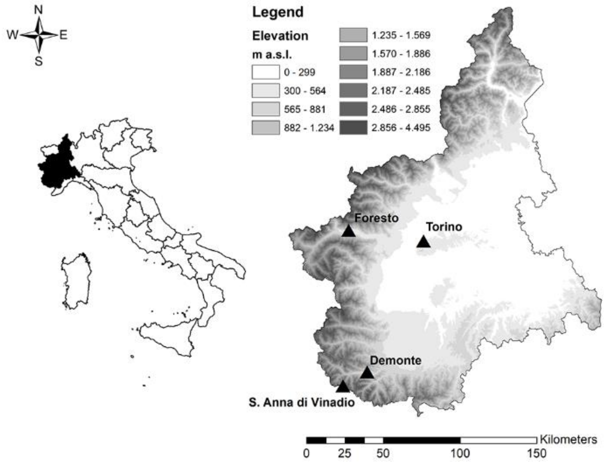

Figure S4 Pairplot of all predictor variables. The upper panel contains estimated pair-wise correlations, and the font size is proportional to the absolute value of the estimated correlation coefficient. The diagonal panel contains histograms and the lower panel scatterplots with a LOESS (locally weighted smoothing) smoother added to aid visual interpretation.

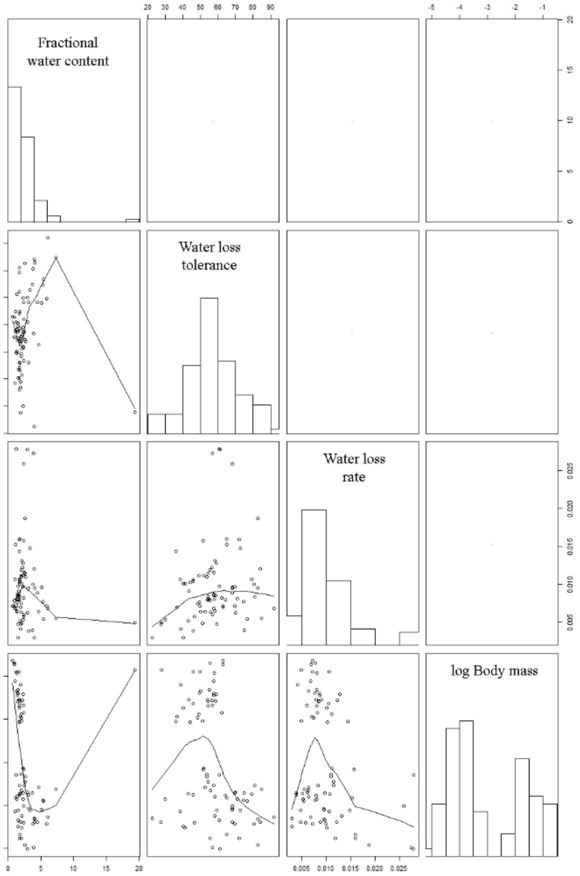

Fractional water content
Water loss tolerance
Water loss rate
log Body mass
