# Supplementary material for: Physiological, morphological and ecological traits drive desiccation resistance in north temperate dung beetles
Source: BMC Zool. 2021 Sep 9;6:26. doi: 10.1186/s40850-021-00089-3 (PMC10127359; doi:10.1186/s40850-021-00089-3)
Supplement: Supplementary file 2 — Additional file 2: Phylogenetic analysis results. Table S1. The variance of the standardized contrasts (VarContr) value define the amount of phylogenetic signal for each trait. Table S2. Independent contrast correlations (traits 2 and higher vs. trait 1). Table S3. Accession codes of the COI-5P sequences used in the phylogenetic analysis. The sequences marked by the asterisk were used in the phylogenetic signal analysis. Box S4. The phylogenetic analysis. Table S5. Maximum Likelihood fits of 24 different nucleotide substitution models. Table S6. Estimates of Evolutionary Divergence between Sequences. Figure S7. The ML tree with the highest log likelihood (-3191.7887). Figure S8. The bootstrap tree from the ML tree with the highest log likelihood (-2731.88). Box S9. The Phylogenetic signal. [file 40850_2021_89_MOESM2_ESM.pdf]

Table S1 The variance of the standardized contrasts (VarContr) value define the amount of phylogenetic signal for each trait. BM, DR, WLR, WLT, fWC, NS are abbreviations that respectively mean Body Mass, Desiccation Resistance, Water Loss Rate, Water Loss Tolerance, Fractional water, Nesting Strategies.

		**Phylogeneti signal**		
**Trait**	**NTaxa**	**VarContr**	**VarCn rankLow**	**VarCn rankHi**
**BM**	8	4341.618	72	928
**DR**	8	0.011	51	949
**WLR**	8	0.000	78	922
**WLT**	8	63.547	480	521
**fWC**	8	9450.207	51	950
**NS**	8	0.357	21	983

Table S2 Independent contrast correlations (traits 2 and higher vs. trait 1). Triangular matrix of the PicR (phylogenetic independent contrasts) values below the diagonal, and triangular matrix of nPos (number of indipendent contrasts) values above the diagonal. BM, DR, WLR, WLT, fWC, NS are abbreviations that respectively mean Body Mass, Desiccation Resistance, Water Loss Rate, Water Loss Tolerance, Fractional water, Nesting Strategies. Here, the total number of contrasts tested (nCont) is 7.

	**BM**	**DR**	**WLT**	**WLR**	**fWC**	**NS**
**BM**		4	0	4	6	3
**DR**	-0.170		3	1	3	6
**WLT**	-0.318	-0.072		3	1	4
**WLR**	-0.061	-0.815	0.523		3	0
**fWC**	0.991	-0.177	-0.308	-0.090		4
**NS**	-0.127	0.666	-0.025	-0.385	-0.144	

Table S3 Accession codes of the COI-5P sequences used in the phylogenetic analysis. The sequences marked by the asterisk were used in the phylogenetic signal analysis.

species	COI sequence accession number	database
Gymnopleurus mopsus (Pallas, 1781)	GBCLS3514-19	BOLD
	GBMIN52740-17	BOLD
Copris lunaris (Linnaeus, 1758)	COLNO266-09	BOLD
	FBCOC373-10	BOLD
	FBCOJ484-12	BOLD
	GBCOB237-12	BOLD
	GBCOU1650-13	BOLD
Trypocopris alpinus (Sturm & Hagenbach, 1825)	FBCOG1297-12	BOLD
Trypocopris vernalis (Linnaeus, 1758)	GBCOB645-12	BOLD
	GCOL2345-16	BOLD
	GCOL3894-16	BOLD
Geotrupes stercorarius (Linnaeus, 1758)	FBCOB57410*	BOLD
Onthophagus taurus (Schreber, 1759)	ICHC02508*	BOLD
	INRMA182614	BOLD
	INRMA182714	BOLD
Onthophagus fracticornis (Preyssler, 1790)	FBCOK35813*	BOLD
	FBCOK51713	BOLD
	GBCOU146313	BOLD
	GCOL132316	BOLD
	GCOL289816	BOLD
Euoniticellus fulvus (Goeze, 1777)	FBCOK51613	BOLD
	GBCLS366919*	BOLD
Sisyphus schaefferi (Linnaeus, 1758)	FBCOK51513*	BOLD
Bodilopsis rufa (Moll, 1782)	COLFF821-13*	BOLD
	FBCOC755-10	BOLD
	FBCOG205-12	BOLD
	GBCOG142-13	BOLD
	GBCOL588-12	BOLD
Rhodaphodius foetens (Fabricius, 1787)	FBCON571-13*	BOLD
	GBCOL601-12	BOLD
	GCOL11973-16	BOLD
Colobopterus erraticus (Linnaeus, 1758)	KU919068.1*	GenBank
	KU918675.1	GenBank
	KU913756.1	GenBank
	KU907735.1	GenBank

Box S4 The phylogenetic analysis

For the phylogenetic analysis, 35 COI-5P sequences (ingroup and outgroup taxa) were obtained from the BOLD and GenBank databases (see **Table S3** for the list of the accession numbers), and aligned using the muscle method by Mega v10 (UPGMB clustering method, Kumar et al., 2018). The dataset was analysed by Maximum Parsimony (MP) and Maximum Likelihood (ML) approaches. In both analyses the nonparametric bootstrapping was performed to assess statistical significance for support of internal nodes (Felsenstein, 1985).

In order to evaluate the best fit model of sequence evolution for the ML analysis, the fit statistics among 24 different nucleotide substitution models were calculated (**Table S5**). The evolutionary divergence between sequences was also estimated, and the number of base substitutions per site with the standard error are given (**Table S6**). The following options were used for the MP heuristic search: search method = TBR; initial trees = 10; max No. trees retained = 100; substitutions type = nucleotide level; bootstrap n reps = 1000. The ML analysis options were set according to the best fit value (**Table S5**) with the substitution model = GTR and rates = G+I; the other options were set as substitutions type = nucleotide level; heuristic method = NNI, with default initial tree; bootstrap n reps = 1000; branch swap filter = very strong. All the trees generated by the analyses showed the same phylogenetic relationships among the taxa. Here the ML tree is presented (**Figure S7**).

For the Phylogenetic Signal (PhylSig) analysis, a reduced matrix was built, in which only a terminal node for each ingroup species was included (**Table S3**, the sequences with the asterisk). No outgroup was included. This reduced matrix was used in the ML analysis, choosing the same options of the former one, since the GTR+G+I model again represents the best fit, being Parameters = 23; BIC = 5660.828; AICc = 5509.964; lnL = -2731.877; (+I) = 0.55; (+G) = 1.62; R = 2.00. In the bootstrap consensus tree inferred from 1000 replicates the branches corresponding to partitions reproduced in less than 50% bootstrap replicates were collapsed. The percentage of replicate trees in which the associated taxa clustered together in the bootstrap test (1000 replicates) are shown next to the branches of the ML tree (**Figure S7**). The analysis gave good bootstrap values (between 95% and 75%) for almost all the nodes, although the phylogenetic position of both *Sisysphus schaefferi* and *Euoniticellus fulvus* is not so clearly defined (bootstrap values below 50%). The bootstrap ML tree was selected for the phylogenetic signal analysis.

References

Felsenstein J. (1985). Confidence limits on phylogenies: An approach using the bootstrap. Evolution 39:783-791.

Kumar S, Stecher G, Li M, Knyaz C, and Tamura K (2018) MEGA X: Molecular Evolutionary Genetics Analysis across computing platforms. Molecular Biology and Evolution 35:1547-1549.

Table S5 Maximum Likelihood fits of 24 different nucleotide substitution models. The models with the lowest BIC scores (Bayesian Information Criterion) are considered to describe the substitution pattern the best. For each model, AICc value (Akaike Information Criterion, corrected), Maximum Likelihood value (lnL), and the number of parameters (including branch lengths) are also presented.

Model	#Param	BIC	AICc	lnL	Invariant	Gamma	R	Freq A	Freq T	Freq C	Freq G	A=>T	A=>C	A=>G	T=>A	T=>C	T=>G	C=>A	C=>T	C=>G	G=>A	G=>T	G=>C
GTR+G+I	77	7145.953	6538.182	-3191.789	0.528	0.931	1.910	0.305	0.371	0.168	0.156	0.130	0.000	0.040	0.110	0.190	0.000	0.000	0.420	0.010	0.080	0.010	0.010
GTR+G	76	7151.876	6551.990	-3199.700	n/a	0.205	1.573	0.305	0.371	0.168	0.156	0.150	0.000	0.050	0.120	0.170	0.000	0.000	0.370	0.010	0.100	0.010	0.010
GTR+I	76	7202.855	6602.969	-3225.190	0.614	n/a	1.610	0.305	0.371	0.168	0.156	0.140	0.010	0.050	0.120	0.170	0.010	0.010	0.380	0.010	0.090	0.020	0.010
TN93+G+I	74	7210.210	6626.096	-3238.769	0.484	0.574	2.426	0.305	0.371	0.168	0.156	0.050	0.020	0.030	0.040	0.200	0.020	0.040	0.440	0.020	0.060	0.050	0.020
TN93+G	73	7212.000	6635.772	-3244.614	n/a	0.162	3.591	0.305	0.371	0.168	0.156	0.040	0.020	0.020	0.030	0.230	0.020	0.030	0.500	0.020	0.040	0.040	0.020
HKY+G	72	7264.053	6695.711	-3275.591	n/a	0.187	1.377	0.305	0.371	0.168	0.156	0.070	0.030	0.090	0.060	0.100	0.030	0.060	0.230	0.030	0.190	0.070	0.030
T92+G	70	7268.411	6715.842	-3287.671	n/a	0.187	1.435	0.338	0.338	0.162	0.162	0.060	0.030	0.100	0.060	0.100	0.030	0.060	0.210	0.030	0.210	0.060	0.030
HKY+G+I	73	7273.954	6697.726	-3275.591	0.000	0.187	1.377	0.305	0.371	0.168	0.156	0.070	0.030	0.090	0.060	0.100	0.030	0.060	0.230	0.030	0.190	0.070	0.030
T92+G+I	71	7278.312	6717.857	-3287.671	0.000	0.187	1.435	0.338	0.338	0.162	0.162	0.060	0.030	0.100	0.060	0.100	0.030	0.060	0.210	0.030	0.210	0.060	0.030
TN93+I	73	7293.950	6717.722	-3285.589	0.624	n/a	0.946	0.305	0.371	0.168	0.156	0.090	0.040	0.050	0.070	0.120	0.040	0.070	0.260	0.040	0.090	0.090	0.040
HKY+I	72	7323.994	6755.652	-3305.562	0.625	n/a	1.257	0.305	0.371	0.168	0.156	0.080	0.030	0.090	0.060	0.100	0.030	0.060	0.220	0.030	0.180	0.080	0.030
T92+I	70	7331.188	6778.619	-3319.059	0.625	n/a	1.263	0.338	0.338	0.162	0.162	0.070	0.030	0.100	0.070	0.100	0.030	0.070	0.200	0.030	0.200	0.070	0.030
K2+G	69	7553.075	7008.393	-3434.954	n/a	0.210	1.331	0.250	0.250	0.250	0.250	0.050	0.050	0.140	0.050	0.140	0.050	0.050	0.140	0.050	0.140	0.050	0.050
K2+G+I	70	7562.976	7010.407	-3434.954	0.000	0.210	1.331	0.250	0.250	0.250	0.250	0.050	0.050	0.140	0.050	0.140	0.050	0.050	0.140	0.050	0.140	0.050	0.050
JC+G	68	7586.125	7049.330	-3456.429	n/a	0.214	0.500	0.250	0.250	0.250	0.250	0.080	0.080	0.080	0.080	0.080	0.080	0.080	0.080	0.080	0.080	0.080	0.080
JC+G+I	69	7596.026	7051.344	-3456.429	0.000	0.214	0.500	0.250	0.250	0.250	0.250	0.080	0.080	0.080	0.080	0.080	0.080	0.080	0.080	0.080	0.080	0.080	0.080
K2+I	69	7600.634	7055.952	-3458.733	0.621	n/a	0.952	0.250	0.250	0.250	0.250	0.060	0.060	0.120	0.060	0.120	0.060	0.060	0.120	0.060	0.120	0.060	0.060
JC+I	68	7636.000	7099.205	-3481.367	0.620	n/a	0.500	0.250	0.250	0.250	0.250	0.080	0.080	0.080	0.080	0.080	0.080	0.080	0.080	0.080	0.080	0.080	0.080
GTR	75	7840.053	7248.053	-3548.740	n/a	n/a	0.805	0.305	0.371	0.168	0.156	0.180	0.040	0.050	0.150	0.110	0.010	0.070	0.250	0.000	0.100	0.020	0.000
TN93	72	7983.153	7414.811	-3635.141	n/a	n/a	0.934	0.305	0.371	0.168	0.156	0.090	0.040	0.040	0.080	0.120	0.040	0.080	0.260	0.040	0.090	0.090	0.040
HKY	71	8016.832	7456.376	-3656.931	n/a	n/a	0.908	0.305	0.371	0.168	0.156	0.090	0.040	0.080	0.080	0.090	0.040	0.080	0.190	0.040	0.150	0.090	0.040
T92	69	8026.930	7482.248	-3671.881	n/a	n/a	0.908	0.338	0.338	0.162	0.162	0.080	0.040	0.080	0.080	0.080	0.040	0.080	0.170	0.040	0.170	0.080	0.040
K2	68	8189.270	7652.476	-3758.002	n/a	n/a	1.128	0.250	0.250	0.250	0.250	0.060	0.060	0.130	0.060	0.130	0.060	0.060	0.130	0.060	0.130	0.060	0.060
JC	67	8212.184	7683.276	-3774.409	n/a	n/a	0.500	0.250	0.250	0.250	0.250	0.080	0.080	0.080	0.080	0.080	0.080	0.080	0.080	0.080	0.080	0.080	0.080

Table S6 Estimates of Evolutionary Divergence between Sequences. The number of base substitutions per site from between sequences are shown below the diagonal, and the standard error estimate(s) are shown above the diagonal.

	GBCLS3514-19	GBMIN52740-17	COLNO266-09	FBCOC373-10	FBCOJ484-12	GBCOB237-12	GBCOU1650-13	FBCOG1297-12	GBCOB645-12	GCOL2345-16	GCOL3894-16	FBCOB57410	ICHC02508	INRMA182614	INRMA182714	FBCOK35813	FBCOK51713	GBCOU146313	GCOL132316	GCOL289816	FBCOK51613	GBCLS366919	FBCOK51513	KU919068.1	KU918675.1	KU913756.1	KU907735.1	COLFF821-13	FBCOC755-10	FBCOG205-12	GBCOG142-13	GBCOL588-12	FBCON571-13	GBCOL601-12	GCOL11973-16
GBCLS3514-19		0.004	0.017	0.017	0.017	0.017	0.017	0.017	0.018	0.018	0.018	0.017	0.016	0.016	0.016	0.018	0.018	0.018	0.017	0.018	0.018	0.018	0.016	0.021	0.021	0.021	0.022	0.017	0.017	0.017	0.017	0.017	0.018	0.018	0.018
GBMIN52740-17	0.009		0.016	0.016	0.016	0.016	0.016	0.017	0.017	0.018	0.018	0.017	0.015	0.015	0.015	0.018	0.018	0.018	0.017	0.018	0.018	0.018	0.016	0.021	0.021	0.021	0.021	0.017	0.017	0.017	0.017	0.017	0.018	0.018	0.018
COLNO266-09	0.153	0.147		0.000	0.000	0.000	0.000	0.019	0.018	0.018	0.018	0.020	0.018	0.018	0.018	0.019	0.020	0.019	0.019	0.019	0.019	0.019	0.019	0.019	0.019	0.019	0.019	0.018	0.018	0.018	0.018	0.018	0.018	0.018	0.018
FBCOC373-10	0.153	0.147	0.000		0.000	0.000	0.000	0.019	0.018	0.018	0.018	0.020	0.018	0.018	0.018	0.019	0.020	0.019	0.019	0.019	0.019	0.019	0.019	0.019	0.019	0.019	0.019	0.018	0.018	0.018	0.018	0.018	0.018	0.018	0.018
FBCOJ484-12	0.153	0.147	0.000	0.000		0.000	0.000	0.019	0.018	0.018	0.018	0.020	0.018	0.018	0.018	0.019	0.020	0.019	0.019	0.019	0.019	0.019	0.019	0.019	0.019	0.019	0.019	0.018	0.018	0.018	0.018	0.018	0.018	0.018	0.018
GBCOB237-12	0.153	0.147	0.000	0.000	0.000		0.000	0.019	0.018	0.018	0.018	0.020	0.018	0.018	0.018	0.019	0.020	0.019	0.019	0.019	0.019	0.019	0.019	0.019	0.019	0.019	0.019	0.018	0.018	0.018	0.018	0.018	0.018	0.018	0.018
GBCOU1650-13	0.153	0.147	0.000	0.000	0.000	0.000		0.019	0.018	0.018	0.018	0.020	0.018	0.018	0.018	0.019	0.020	0.019	0.019	0.019	0.019	0.019	0.019	0.019	0.019	0.019	0.019	0.018	0.018	0.018	0.018	0.018	0.018	0.018	0.018
FBCOG1297-12	0.155	0.147	0.186	0.186	0.186	0.186	0.186		0.013	0.013	0.013	0.015	0.019	0.019	0.019	0.019	0.019	0.019	0.019	0.019	0.017	0.017	0.017	0.020	0.020	0.020	0.020	0.019	0.019	0.019	0.019	0.019	0.020	0.020	0.020
GBCOB645-12	0.171	0.160	0.164	0.164	0.164	0.164	0.164	0.090		0.007	0.007	0.017	0.018	0.018	0.018	0.018	0.019	0.018	0.018	0.018	0.019	0.018	0.018	0.020	0.020	0.020	0.021	0.020	0.020	0.020	0.020	0.020	0.019	0.019	0.019
GCOL2345-16	0.178	0.167	0.169	0.169	0.169	0.169	0.169	0.092	0.023		0.002	0.017	0.018	0.018	0.018	0.019	0.019	0.018	0.018	0.019	0.019	0.019	0.018	0.021	0.021	0.021	0.022	0.020	0.020	0.020	0.020	0.020	0.020	0.020	0.020
GCOL3894-16	0.180	0.169	0.171	0.171	0.171	0.171	0.171	0.090	0.025	0.002		0.018	0.018	0.018	0.018	0.019	0.019	0.018	0.018	0.019	0.019	0.019	0.018	0.021	0.021	0.021	0.021	0.020	0.020	0.020	0.020	0.020	0.020	0.020	0.020
FBCOB57410	0.168	0.164	0.191	0.191	0.191	0.191	0.191	0.130	0.167	0.172	0.174		0.018	0.018	0.018	0.019	0.019	0.019	0.018	0.019	0.018	0.018	0.017	0.021	0.021	0.021	0.021	0.021	0.021	0.021	0.021	0.021	0.019	0.019	0.019
ICHC02508	0.153	0.147	0.168	0.168	0.168	0.168	0.168	0.173	0.160	0.164	0.167	0.173		0.000	0.000	0.016	0.016	0.016	0.016	0.016	0.018	0.018	0.016	0.020	0.020	0.020	0.020	0.018	0.018	0.018	0.018	0.018	0.019	0.019	0.019
INRMA182614	0.153	0.147	0.168	0.168	0.168	0.168	0.168	0.173	0.160	0.164	0.167	0.173	0.000		0.000	0.016	0.016	0.016	0.016	0.016	0.018	0.018	0.016	0.020	0.020	0.020	0.020	0.018	0.018	0.018	0.018	0.018	0.019	0.019	0.019
INRMA182714	0.153	0.147	0.168	0.168	0.168	0.168	0.168	0.173	0.160	0.164	0.167	0.173	0.000	0.000		0.016	0.016	0.016	0.016	0.016	0.018	0.018	0.016	0.020	0.020	0.020	0.020	0.018	0.018	0.018	0.018	0.018	0.019	0.019	0.019
FBCOK35813	0.168	0.160	0.177	0.177	0.177	0.177	0.177	0.173	0.173	0.185	0.182	0.182	0.134	0.134	0.134		0.003	0.002	0.004	0.000	0.018	0.018	0.016	0.019	0.019	0.019	0.019	0.020	0.020	0.020	0.020	0.020	0.018	0.018	0.018
FBCOK51713	0.166	0.162	0.182	0.182	0.182	0.182	0.182	0.175	0.175	0.189	0.187	0.186	0.138	0.138	0.138	0.007		0.004	0.005	0.003	0.018	0.018	0.017	0.019	0.019	0.019	0.019	0.019	0.019	0.019	0.019	0.019	0.018	0.018	0.018
GBCOU146313	0.168	0.160	0.175	0.175	0.175	0.175	0.175	0.175	0.169	0.180	0.178	0.180	0.134	0.134	0.134	0.004	0.011		0.004	0.002	0.018	0.018	0.016	0.019	0.019	0.019	0.019	0.020	0.020	0.020	0.020	0.020	0.017	0.017	0.017
GCOL132316	0.162	0.153	0.173	0.173	0.173	0.173	0.173	0.168	0.169	0.175	0.173	0.177	0.128	0.128	0.128	0.009	0.012	0.009		0.004	0.018	0.018	0.017	0.019	0.019	0.019	0.019	0.019	0.019	0.019	0.019	0.019	0.018	0.018	0.018
GCOL289816	0.168	0.160	0.177	0.177	0.177	0.177	0.177	0.173	0.173	0.185	0.182	0.182	0.134	0.134	0.134	0.000	0.007	0.004	0.009		0.018	0.018	0.016	0.019	0.019	0.019	0.019	0.020	0.020	0.020	0.020	0.020	0.018	0.018	0.018
FBCOK51613	0.167	0.160	0.171	0.171	0.171	0.171	0.171	0.158	0.187	0.194	0.191	0.173	0.169	0.169	0.169	0.173	0.175	0.175	0.173	0.173		0.004	0.017	0.018	0.018	0.018	0.018	0.018	0.018	0.018	0.018	0.018	0.018	0.018	0.018
GBCLS366919	0.173	0.167	0.173	0.173	0.173	0.173	0.173	0.162	0.184	0.191	0.189	0.178	0.173	0.173	0.173	0.177	0.180	0.175	0.173	0.177	0.011		0.017	0.018	0.018	0.018	0.018	0.018	0.018	0.018	0.018	0.018	0.018	0.018	0.018
FBCOK51513	0.155	0.153	0.184	0.184	0.184	0.184	0.184	0.151	0.169	0.178	0.181	0.163	0.156	0.156	0.156	0.153	0.162	0.153	0.160	0.153	0.153	0.160		0.020	0.020	0.020	0.020	0.019	0.019	0.019	0.019	0.019	0.018	0.018	0.018
KU919068.1	0.212	0.202	0.176	0.176	0.176	0.176	0.176	0.193	0.207	0.222	0.219	0.205	0.180	0.180	0.180	0.173	0.175	0.171	0.173	0.173	0.171	0.173	0.187		0.000	0.000	0.002	0.016	0.016	0.016	0.016	0.016	0.018	0.018	0.018
KU918675.1	0.212	0.202	0.176	0.176	0.176	0.176	0.176	0.193	0.207	0.222	0.219	0.205	0.180	0.180	0.180	0.173	0.175	0.171	0.173	0.173	0.171	0.173	0.187	0.000		0.000	0.002	0.016	0.016	0.016	0.016	0.016	0.018	0.018	0.018
KU913756.1	0.212	0.202	0.176	0.176	0.176	0.176	0.176	0.193	0.207	0.222	0.219	0.205	0.180	0.180	0.180	0.173	0.175	0.171	0.173	0.173	0.171	0.173	0.187	0.000	0.000		0.002	0.016	0.016	0.016	0.016	0.016	0.018	0.018	0.018
KU907735.1	0.214	0.205	0.178	0.178	0.178	0.178	0.178	0.195	0.210	0.224	0.222	0.205	0.183	0.183	0.183	0.175	0.178	0.173	0.175	0.175	0.173	0.175	0.189	0.002	0.002	0.002		0.017	0.017	0.017	0.017	0.017	0.018	0.018	0.018
COLFF821-13	0.158	0.155	0.184	0.184	0.184	0.184	0.184	0.175	0.201	0.201	0.203	0.195	0.154	0.154	0.154	0.189	0.187	0.191	0.187	0.189	0.163	0.167	0.181	0.141	0.141	0.141	0.143		0.000	0.000	0.000	0.000	0.018	0.018	0.018
FBCOC755-10	0.158	0.155	0.184	0.184	0.184	0.184	0.184	0.175	0.201	0.201	0.203	0.195	0.154	0.154	0.154	0.189	0.187	0.191	0.187	0.189	0.163	0.167	0.181	0.141	0.141	0.141	0.143	0.000		0.000	0.000	0.000	0.018	0.018	0.018
FBCOG205-12	0.158	0.155	0.184	0.184	0.184	0.184	0.184	0.175	0.201	0.201	0.203	0.195	0.154	0.154	0.154	0.189	0.187	0.191	0.187	0.189	0.163	0.167	0.181	0.141	0.141	0.141	0.143	0.000	0.000		0.000	0.000	0.018	0.018	0.018
GBCOG142-13	0.158	0.155	0.184	0.184	0.184	0.184	0.184	0.175	0.201	0.201	0.203	0.195	0.154	0.154	0.154	0.189	0.187	0.191	0.187	0.189	0.163	0.167	0.181	0.141	0.141	0.141	0.143	0.000	0.000	0.000		0.000	0.018	0.018	0.018
GBCOL588-12	0.158	0.155	0.184	0.184	0.184	0.184	0.184	0.175	0.201	0.201	0.203	0.195	0.154	0.154	0.154	0.189	0.187	0.191	0.187	0.189	0.163	0.167	0.181	0.141	0.141	0.141	0.143	0.000	0.000	0.000	0.000		0.018	0.018	0.018
FBCON571-13	0.180	0.173	0.184	0.184	0.184	0.184	0.184	0.193	0.198	0.198	0.201	0.173	0.183	0.183	0.183	0.160	0.169	0.155	0.160	0.160	0.167	0.173	0.167	0.161	0.161	0.161	0.163	0.161	0.161	0.161	0.161	0.161		0.000	0.000
GBCOL601-12	0.180	0.173	0.184	0.184	0.184	0.184	0.184	0.193	0.198	0.198	0.201	0.173	0.183	0.183	0.183	0.160	0.169	0.155	0.160	0.160	0.167	0.173	0.167	0.161	0.161	0.161	0.163	0.161	0.161	0.161	0.161	0.161	0.000		0.000
GCOL11973-16	0.180	0.173	0.184	0.184	0.184	0.184	0.184	0.193	0.198	0.198	0.201	0.173	0.183	0.183	0.183	0.160	0.169	0.155	0.160	0.160	0.167	0.173	0.167	0.161	0.161	0.161	0.163	0.161	0.161	0.161	0.161	0.161	0.000	0.000	

Figure S7 The ML tree with the highest log likelihood (-3191.7887) is shown. The ML tree was obtained by the GTR (General Time Reversible) model. A discrete Gamma distribution was used to model evolutionary rate differences among sites (5 categories (+G, parameter = 0.9309)). The rate variation model allowed for some sites to be evolutionarily invariable ([+I], 52.8325% sites). The tree is drawn to scale, with branch lengths measured in the number of substitutions per site. The tree was rooted by the outgroup method, using the *Trypocopis* species as outgroup.

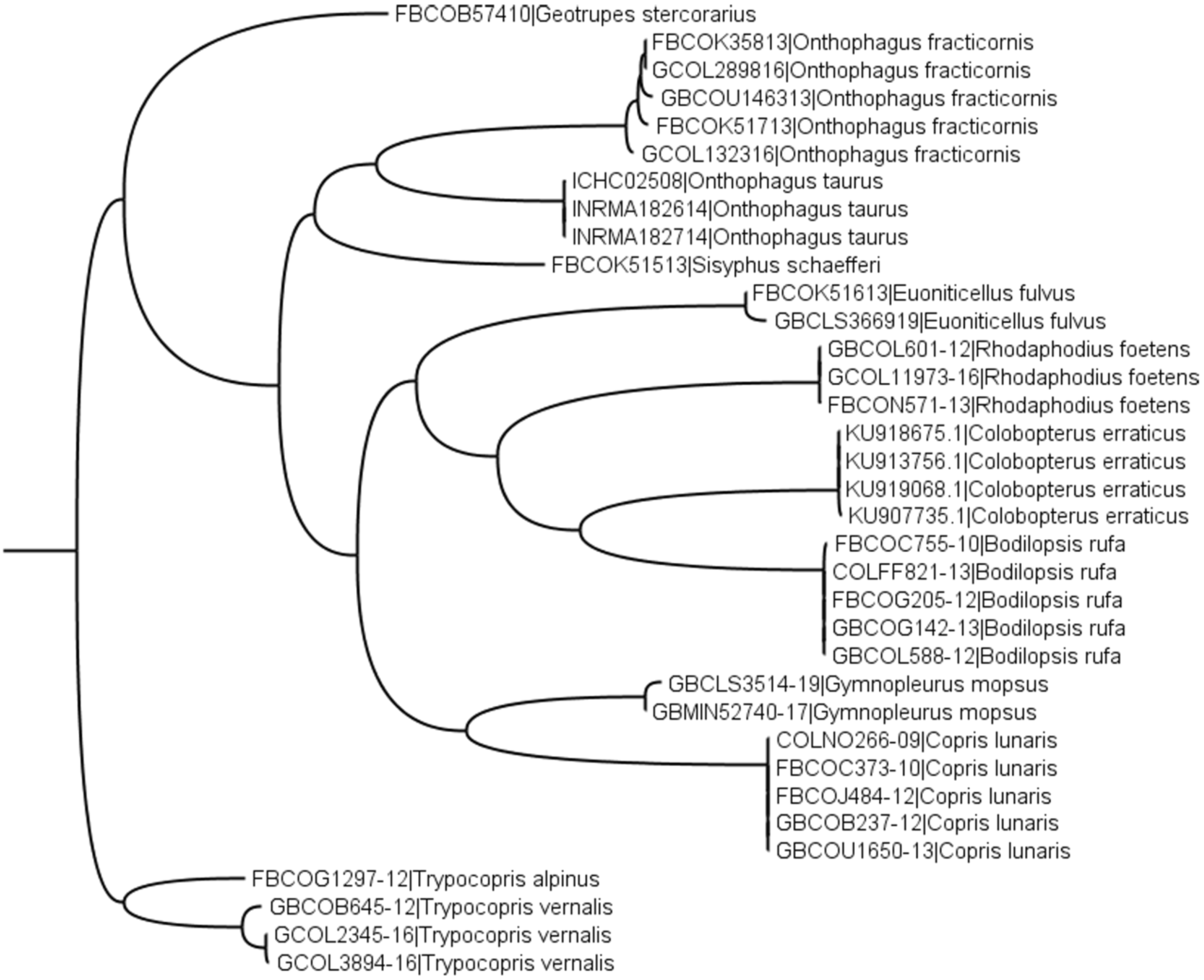

Figure S8 The bootstrap tree from the ML tree with the highest log likelihood (-2731.88); on the tree the numbers of the internal nodes were added (in red). The evolutionary history was inferred by using the Maximum Likelihood method, GTR model. The percentage of trees in which the associated taxa clustered together is shown next to the branches (in black). Here, all the branches are showed as fully resolved. A discrete Gamma distribution was used to model evolutionary rate differences among sites, with 5 categories (+G, parameter = 1.62). The rate variation model allowed for some sites to be evolutionarily invariable ([+I], 55% sites).

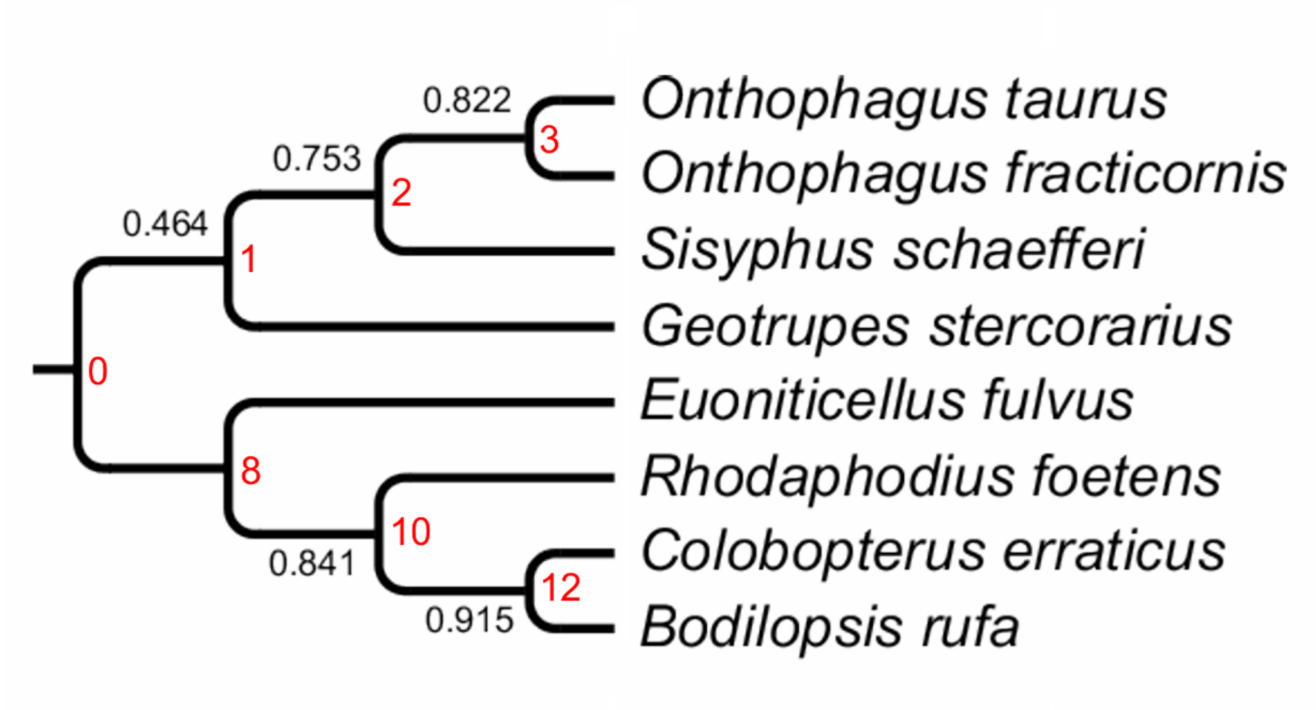

Box S9 The Phylogenetic signal

The software PhyloCom v4.2 (Webb et al., 2008) was used to define the phylogenetic signal of the traits. A matrix of the mean values of each trait for each species and the reduced ML tree (Fig A4) were used to correlate the eight species and and six trais (i.e, desiccation resistance [DR], dry body mass [BM], nesting strategy [NS], water loss rate [WLR], water loss tolerance [WLT], and initial water content [iWC]), based on the phylogenetic relationships of the taxa.

In the traits file the desiccation resistance, dry body mass, water loss rate, water loss tolerance, and initial water content were coded as continuous data, while the functional group was coded as ordered multistate. The AOT module was used to conduct the test of phylogenetic signal (= the tendency for close relatives to resemble each other) and traits correlations.

The variance of the standardized contrasts (VarContr, in Supplemental Material, **Table S1**) values were used as a measure of the phylogenetic signal, being the smaller values an index of stronger dependence of the trait, as is here the case for WRL and DR. If related species are similar to each other, a small variance of contrast values will results, since the magnitude of independent contrasts will be similar across the tree. In this framework, the VarContr values for BM and iWC vouch for a very great variance for both traits.

The evolutionary correlations between traits were calculated by using independent contrasts defining in turn each trait as the independent variable (traitX) and thence all the others as the dependent variable (traitY). The significance testing of the analysis was conducted by randomization (N = 9999 by default). The traits were analysed in pairs, and the significance of the resulting correlations (**Table S2**) can be evaluated using the tables of critical values for the Pearson correlation coefficient (df = 6). According to the PicR (phylogenetic independent contrasts) values, only the pair iWC/BM (R = 0.991, p = 0.001) and DR/NS (R = 0.666, p = 0.1) are significantly correlated. The other pairs showed instead different degree di negative correlation, being the pair WLR/DR (R = -0.815) almost wholly uncorrelated.

References

Webb, C. O., Ackerly, D. D., and Kembel, S. W. 2008. Phylocom: software for the analysis of phylogenetic community structure and character evolution. Bioinformatics 24: 2098-2100.
